# Report on the 2nd scientific meeting of the "Verein zur Förderung des Wissenschaftlichen Nachwuchses in der Neurologie" (NEUROWIND e.V.) held in Motzen, Germany, Oct. 29'th - Oct. 31'st, 2010

**DOI:** 10.1186/2040-7378-3-3

**Published:** 2011-04-01

**Authors:** Tim Magnus, Ralf A Linker, Sven G Meuth, Christoph Kleinschnitz, Thomas Korn

**Affiliations:** 1 Department of Neurology, University Clinic Hamburg-Eppendorf, Martinistr. 52,20246 Hamburg, Germany; 2 Department of Neurology, Friedrich-Alexander-Universität Erlangen, Schwabachanlage 6,91054 Erlangen, Germany; 3 Department of Neurology-Inflammatory Disorders of the Nervous System and Neuro-oncology, Westfälische Wilhelms-University Münster, Domagkstr. 13,48149 Münster, Germany; 4 Department of Neurology, Julius-Maximilians University of Würzburg, Josef-Schneider-Str. 11,97080 Würzburg, Germany; 5 Department of Neurology, Klinikum rechts der Isar, Technical University Munich, Ismaninger Str. 22,81675 Munich, Germany

## Summary of the scientific contributions to the NEUROWIND meeting 2010: Contributions in the fields of neuroimmunology and neurodegeneration

T cell driven autoimmune inflammation in the CNS has widely been investigated in the model of experimental autoimmune encephalomyelitis (EAE) [1]. During decades of EAE research, it has been established that autoreactive T cells are activated in the peripheral immune tissue, then enter the CNS compartment and - upon local re-activation - acquire the ability to invade the CNS parenchyma and exert effector functions. Only with the advent of modern imaging techniques has it become possible to actually visualize the individual steps of T cell activation in the lymph nodes, of crossing the blood brain barrier, and of interaction between autoreactive T cells and their molecular targets within the CNS. Alexander Flügel has adapted the model of adoptive transfer EAE for imaging purposes making inflammatory processes accessible to two-photon-microscopy in situ. By retroviral expression of fluorescent proteins in encephalitogenic T cells, these T cells were visualized in vivo by two photon microscopy [2]. Christian Schläger from Alexander Flügel's group showed two-photon scanning data providing evidence that in the CNS vasculature encephalitogenic T cells tended to crawl against the blood stream before they left the vessel lumen in order to enter the perivascular space. Here, the cells appeared to be scanning their environment and only upon productive contact with antigen presenting cells that presented the appropriate antigen, T cells were instructed to infiltrate into the CNS parenchyma. It is becoming increasingly clear that many features of leukocyte extravasation in the CNS vasculature are unique and distinct from leukocyte extravasation in other vascular territories [3]. The advanced imaging tools that are now available hold promise to address current questions of T lymphocyte biology at the blood brain barrier: Why do lymphocytes move against the blood stream in the CNS microvasculature? Do lymphocytes trespass the endothelial barrier in a paracellular or transcellular way? How and to what extent do T cells become activated in the perivascular space?

The technology of two-photon-microscopy even allows monitoring immune cell-target interactions within the CNS parenchyma. In a recently published study, Volker Siffrin from the group of Frauke Zipp investigated the interaction of encephalitogenic CD4^+ ^T cells with neuronal structures in the brain stem in vivo. Interestingly, myelin antigen reactive (2D2) T cells of the Th17 phenotype were able to interact with (and damage) axons. While IFN-γ producing Th1 cells failed to induce neuronal apoptosis, Th17 cells were very efficient in promoting axonal damage. The mechanism of lesion development has not yet been entirely unraveled. While the interaction between CD4^+ ^T cells and axons was independent of the T cell receptor (which in this case was MOG_35-55 _specific) and thus not restricted by MHC class II expression on axons, ICAM-1 expression by axons and LFA-1 expression by T cells was critically required for Th17-axonal interaction. Axons responded to Th17 cell-mediated attack by Ca^2+ ^influx, which was partially reversible by blockade of NMDA receptors[4]. Thus, Th17 cells exerted effector functions in the CNS that appeared to be unique to this effector T cell subset.

In order to test the functional relevance of susceptibility genes identified in the genome-wide association studies in MS, it is a promising approach to investigate whether the expression level of the corresponding gene products on T cells correlates with an altered functional phenotype of these cells. Melanie Piedavent from the group of Manuel Friese analysed the expression of CD226 on human and mouse CD4^+ ^and CD8^+ ^T cells. CD226 interacts with its ligand CD155 on antigen presenting cells and has a role as a costimulatory molecule. The nonsynonymous single nucleotide polymorphism (SNP) rs763361/Gly307Ser in exon 7 of CD226 leads to the substitution of serine for glycine in the amino acid sequence of CD226 and has been associated with increased risk for type 1 diabetes, MS, rheumatoid arthritis and autoimmune thyroid disease [5]. Both in mouse and in human CD4^+ ^T cells, low and high expression of CD226 segregated with markers of naive and antigen experienced/memory T cells, respectively. CD8^+ ^T cells expressed high amounts of CD226 in a constitutive manner. The functional consequences of rs763361/Gly307Ser are not known. It is possible that the amino acid substitution at position 307 alters the phosphorylation sites of CD226 at positions 322 and 329. Alternatively, an altered expression pattern of CD226 could be induced. Using a mouse model, the functional consequences of Gly307Ser can now be tested in vivo.

The role of γδ T cells in EAE has recently been investigated in more detail. γδ T cells have drawn attention since a subset of γδ T cells was identified to be highly responsive to IL-23, which is known to be a potent driver of autoimmunity and chronic inflammation. Thus, the role of γδ T cells in models of autoimmunity has been revisited. Franziska Petermann from the group of Thomas Korn could show that γδ T cells that respond to IL-23 were very efficient in inhibiting Treg responses. As a result, adaptive immune responses flared up in a milieu that was enriched in IL-23R^+^γδ T cells [6]. While the mechanism of this particular function of γδ T cells has to be further investigated, the role of IL-23R^+^γδ T cells in restraining Treg responses was clinically relevant. Tcrd KO mice that lack γδ T cells had exaggerated Treg responses. Conversely, deletion of Tregs in Tcrd KO mice restored full susceptibility to EAE [6].

In addition to T cells, B cells are increasingly recognized as important players in neuroimmunological diseases. This concept is also supported by the therapeutic efficacy of the B cell depleting anti CD20 antibody rituxan in neuroimmunological disorders. Miguel Maurer from the group of Jan Lünemann, Zurich analyzed the B cell repertoire after rituxan treatment of anti-myelin associated glycoprotein (MAG) antibody positive paraproteinemic neuropathy, an autoimmune disorder of the peripheral nervous system characterized by the presence of antibodies against myelin associated glycoprotein MAG. Rituxan did not influence the B cell receptor repertoire, but reduced clonal expansions of IgM positive memory B cells with reactivity against MAG protein.

The EAE model is an excellent model to investigate T cell development and T cell regulation in vivo. However, the role of antibodies in autoimmune neuroinflammation is not well addressed in classical MOG_35-55 _induced EAE. Moreover, since in MS the most relevant autoantigen is still unknown, the function of EAE as a model for MS is limited in various aspects. On the other hand, a considerable body of knowledge has emerged in the recent years on the target antigen in neuromyelitis optica (NMO) which has been regarded as a variant of MS. However, NMO is probably a distinct disease because the target autoantigen is aquaporin-4 (AQP4) which is not a myelin antigen. AQP4 is a water channel protein which is expressed in astrocytic endfeet of the lamina gliae limitans and thus plays an important part in the function of the blood/brain- and CSF/brain-barriers [7]. Antibodies to AQP4 (NMO-IgG) have been identified in sera of NMO patients and were proven to be a useful biomarker for NMO since NMO-IgG are negative in MS patients [8,9]. NMO-IgG have now been included in the diagnostic criteria for NMO [10]. However, it has still been unclear whether antibodies to AQP4 that are usually not produced intrathecally are pathogenetically relevant. Several laboratories have designed experiments in order to test whether NMO-IgG was able to induce damage to astrocytes [11-13]. In one approach, which was presented by Claudia Wrzos from the group of Christine Stadelmann, a subclinical EAE was induced in experimental rats by active immunization with MBP_72-85 _in CFA followed by intravenous transfer of either control IgG or recombinant monoclonal anti-AQP4 IgG. Recombinant anti-AQP4 antibodies were engineered from heavy and light chain genes isolated from intrathecal plasma cells of NMO patients. The heavy and light chain genes were cloned into human framework cassettes and expressed in HEK293 cells. Recombinant immunoglobulins recognized both the M1 and M23 translational isoform of AQP4. Only when rats received anti-AQP4 antibodies, they show astrocytic damage, Ig deposition, and complement activation at the blood brain barrier in histopathological analyses. In a second approach, recombinant anti-AQP4 antibodies were injected intrathecally together with human complement. Here, astrocyte loss was detected as early as 1 h after injection and oligodendrocyte apoptosis (NogoA^+^caspase-3^+^) as early as 3 h after injection. These experiments were among the first to suggest that NMO-IgG might have pathogenic relevance beyond their great value as biomarker. Thus, astrocytes at the blood brain barrier appear to be a prime target of the inflammatory process in NMO.

The blood brain barrier (BBB) can be the primary target of an autoimmune reaction - as in NMO. However, the blood brain barrier is also crucial in modulating the pathogenic process in a series of inflammatory, ischemic, and degenerative diseases. Therefore, it is essential to understand the function of the BBB in health and disease. In the laboratory of Sven Meuth, it was found that a member of the two-pore domain potassium channel family (K_2P_), namely TWIK-related K^+^-channel gene (TREK-1) is expressed on murine and human endothelial cells. Inhibition of channel activity by pharmacological strategies or during inflammation was associated with a decreased transendothelial resistance (TER) in an in vitro model of the BBB. Activation of channel activity resulted in increased TER and decreased transmigration of immune cells in the same model. Translated to an in vivo model Stefan Bittner demonstrated an enhanced EAE disease course in TREK^-/- ^mice after MOG_35-55 _immunization while activation of the channel in vivo using riluzole and/or α-linolenic acid resulted in a significantly ameliorated EAE phenotype with reduced cellular infiltrates.

Dirk Hermann presented data on the regulation of luminal and abluminal ATP binding cassette transporters in CNS endothelial cells. ABCB1 is expressed in the luminal membrane and ABCC1 in the abluminal membrane. Upon ischemia, ABCB1 was up-regulated, while ABCC1 was down-regulated suggesting that the efflux of xenobiotics out of ischemic brain regions was facilitated while the influx of molecules using the ABC transporter system would be severely inhibited [14]. Interestingly, ApoE mediated the regulation of ABC transporters in the luminal and abluminal membranes via ApoE receptor 2 and the deactivation of JNK1/2 by dephosphorylation. As a consequence, ApoE KO mice showed decreased expression of ABCB1 and increased expression of abluminal ABCC1 upon ischemic brain injury. Thus, modulation of the ABC system appears to be possible by targeting ApoE which has the role of a molecular switch. This system could be exploited to facilitate the delivery of neuroprotective drugs into ischemic brain regions.

In addition to the investigation of immune cells and the blood brain barrier, studies on the target cells of the nervous system have been in the focus of interest in neuroimmunological research. To investigate the role of cells of the oligodendrocyte lineage, Karin Hagemeier from Tanja Kuhlmann`s group in Muenster presented a new co-culture system with primary oligodendrocyte precursor cells and retinal ganglion cells allowing for the analysis of oligodendrocyte - neuron interaction on a single animal basis with high cell purity. In particular, a focus of interest has been on factors influencing cell death of oligodendrocytes. Here, p53 induced pro-apoptotic member of the Bcl2 family (PUMA) is an interesting candidate and the analysis of PUMA deficient cells in culture and PUMA deficient mice in the cuprizone model of de- and remyelination in vivo will reveal the role of this factor in the regulation of oligodendrocyte survival.

In order to understand the role of microglial cells in remyelination, the cuprizone model was also investigated in Martin Stangel's laboratory. During 6 weeks of cuprizone feeding, toxic demyelination is induced in the absence of blood brain barrier breakdown. Remyelination occurs when cuprizone feeding is stopped and takes 6 weeks to be completed. Demyelination is preceded by microglia activation and proliferation by 2 weeks, and remyelination is preceded by proliferation of oligodendrocyte precursor cells (OPC). Thomas Skripuletz from Martin Stangel's laboratory realized that administration of LPS modulated both de- and remyelination in the cuprizone model. The net effect of LPS was beneficial because demyelination was decelerated and remyelination was enhanced. In histological analyses, the proliferation of microglial cells seemed to be inhibited while the proliferation of OPCs was increased by LPS. Thus, both de- and remyelination are modulated by TLR ligands in an indirect manner. However, it remains to be determined whether the decreased proliferation of microglia by LPS, which is able to cross the intact BBB, is the direct cause of decreased demyelination in this model.

In view of the degenerative changes in autoimmune demyelination, neuroprotective approaches are of high interest in MS therapy. De-Hyung Lee from the group of Ralf Linker, Erlangen, presented data on mechanisms of action of fumaric acid esters (FAE), which are currently under investigation as new oral disease modifying drug in relapsing remitting MS. Application of FAE in the MOG-EAE model resulted in an ameliorated course of chronic EAE and a preservation of neurons, oligodendrocytes and myelin as well as reduced astrogliosis without direct influence on the immune reaction. These neuroprotective effects were associated with the activation of nuclear factor (erythroid-derived 2)-like 2 (Nrf2), a transcription factor involved in the cellular detoxification pathways and the natural antioxidative response [15].

Finally, degenerative processes in autoimmune demyelination share several features with primary neurodegenerative diseases like amyotrophic lateral sclerosis (ALS) or Alzheimer's disease (AD). Sarah Kaiser from the Department of Neurology, University Hospital Ulm, presented data on several cerebrospinal fluid (CSF) biomarkers in ALS. The group of Johannes Brettschneider could show that SMI 35 (representing heavy neurofilaments) was increased in rapidly progressing ALS. In contrast, CSF levels of soluble amyloid precursor protein (sAPP) did not show differences to controls, but negatively correlated with disease duration. In conclusion, the NFH/sAPP ratio may represent a new biomarker for ALS progression and may also be of interest in diseases like MS.

The intimate mechanistic relationship between neurodegenerative and neuroinflammatory disease processes is further highlighted by the immune reaction in AD which particularly involves microglial activation. Here, Marius Krauthausen from the group of Marcus Mueller, Bonn, gave an update on their studies on chemokines in the transgenic APP/PS1 model of AD. They investigated the role of CXCR3 in a genetic approach by crossing APP/PS1 mice with CXCR3-deficient mice. As compared to APP/PS1 transgenic controls, these mice display a reduced plaque burden and reduced Aβ protein load associated with a reduced activation and accumulation of periplaque microglia. These data argue for a role of chemokines in plaque formation which may be critically modulated by the function of microglia.

So far, therapeutic options in neurodegenerative diseases are limited. Recently, the application of small inhibitory RNA (siRNA) came into the focus of interest. Anderson de Andrade and Xu Hong, both from Guenter Hoeglinger's group in Marburg, employed siRNA as new therapeutic approach in tauopathies and synucleinopathies. In vitro models using adenoviral overexpression of alpha synuclein in dopaminergic neurons and the in vivo model of P301S tau transgenic mice will allow for refined protocols of siRNA application and thus testing the effect of synuclein or tau knock-down as new therapeutic approaches in neurodegenerative diseases.

## Contributions on stroke and vascular pathology

In the stroke field, the role of the immune system was discussed. First studies show that specific inhibition of sphingolipid signaling or inhibition of adhesion molecules can be beneficial. Waltraud Pfeilschifter from Frankfurt presented data that treatment with FTY720, a functional sphingosin 1 phosphate receptor 1 antagonist which blocks the egress of lymphocytes from the lymph node, reduced ischemic damage in the middle cerebral artery occlusion (MCAO) model. It also reduced the activation of the immune system and apoptotic cell death. Arthur Liesz from Heidelberg demonstrated the potential of interfering with the migration of leucocytes across the blood brain barrier by inhibition of VCAM through antibody or siRNA[16]. Especially, the gene silencing resulted in a better outcome in the MCAO model. Furthermore, Friederike Vollmar from the group of Christoph Kleinschnitz in Wuerzburg highlighted novel findings on the role of the proinflammatory kallikrein-kinin-system (KKS) in the pathophysiology of acute ischemic stroke. As previously shown, depletion or pharmacological blockade of the bradykinin receptor B1 (B1R), but not B2R, attenuated postischemic inflammation and blood-brain-barrier damage both after transient middle cerebral artery occlusion or traumatic brain injury in mice [17,18]. In a follow-up project, the group currently investigates whether additional molecules located upstream of the kinin receptors such as kininogen or plasma kallikrein are likewise involved in stroke-induced inflammation and neuronal damage. Preliminary data obtained in kininogen(kng)-deficient mice were indicative for a potential role of KNG for thrombus formation and edema formation in the ischemic brain.

However, the immune regulation in stroke is quite complex and involves different subclasses of immune cells. Mathias Gelderblom from the group of Tim Magnus showed that three days following stroke γδ T cells emerged in the ischemic hemisphere. The majority of γδ T cells were located in direct proximity to the infarct core. 40% of these atypical T cells produced IL 17A and seemed to have a role in recruiting neutrophils to the area of destruction. The complexity of the immunologic reperfusion response after stroke and possible pitfalls in immune cell depletion approaches as a potential therapeutic strategy were further underscored by the observation of Michael Gliem from Sebastian Jander's group showing that depletion of macrophages with clodronate resulted in an increased bleeding rate after MCAO.

The peptide hormone Ghrelin is known as the ligand of the growth hormone secretagogue receptor. Ghrelin crosses the blood-brain barrier and binds to hippocampal neurons thereby promoting dendritic spine synapse formation and proliferation of progenitor cells. Kai Diederich together with Jens Minnerup from Münster demonstrated that Ghrelin treatment improves functional recovery after photothrombotic stroke in rats probably by enhancing the generation of newborn hippocampal neurons.

Felix Schlachetzki from Regensburg reviewed basic mechanisms of blood-brain barrier (BBB) damage following brain ischemia/reperfusion injury which is associated with intracerebral hemorrhage and edema formation. He pointed out that in experimental stroke BBB permeability is bi-phasic for certain contrast agents (para-endothelial efflux) yet vasogenic edema is a monophasic event (trans-endothelial efflux) as shown by serial post-contrast MRI and T2-relaxometry. However, the bi-phasic BBB response may be linked to both deleterious and regenerative effects at the neurovasular unit [19].

In conclusion, by bringing together researchers in the fields of neuroimmunology, neurodegeneration, and neurovascular diseases, this meeting has again been a valuable platform to discuss pathogenic cascades common to these different disorders. Access of immune cells (innate or adaptive) to different body compartments and in particular to the CNS are clearly common themes in a variety of neurological diseases. It is our hope that the NEUROWIND meeting will teach us how it might be possible to advance the understanding of pathogenic processes in neurological disorders by exchanging concepts and tools between various CNS disease models.

## Competing interests

The authors declare that they have no competing interests.

## Authors' contributions

TM, RL, SGM, CK, and TK wrote the paper. All authors read and approved the final manuscript.

## List of speakers at the second scientific meeting of NEUROWIND e.V. (in alphabetical order)

Anderson de Andrade, Dept. of Neurology, University of Marburg, Germany

Stefan Bittner, Dept. of Neurology, University of Münster, Germany

Kai Diederich, Dept. of Neurology, University of Münster, Germany

Ulrich Dirnagl, Dept. of Neurology and Experimental Neurology and Center for Stroke Research, Berlin Charité University Medicine, Germany

Mathias Gelderblom, Center for Molecular Neurobiology, Hamburg, Germany

Michael Gliem, Dept. of Neurology, University of Düsseldorf, Germany

Karin Hagemeier, Dept. of Neurology, University of Münster, Germany

Dirk Hermann, Dept. of Neurology, University of Essen, Germany

Sarah Kaiser, Dept. of Neurology, University of Ulm, Germany

Marius Krauthausen, Dept. of Neurology, University of Bonn, Germany

De-Hyung Lee, Dept. of Neurology, University of Bochum, Germany

Arthur Liesz, Dept. of Neurology, University of Heidelberg, Germany

Miguel Maurer, Institute for Experimental Neurology, University of Zürich, Switzerland

Philipp Mergenthaler, Charité University Medicine, Berlin, Germany

Franziska Petermann, Dept. of Neurology, Technical University of Munich, Germany

Waltraud Pfeilschifter, Dept. of Neurology, University of Frankfurt, Germany

Melanie Piedavent, Center for Molecular Neurobiology, Hamburg, Germany

Franziska Scheibe, Dept. of Experimental Neurology, Berlin Charité University Medicine, Germany

Felix Schlachetzki, Dept. of Neurology, University Regensburg, Germany

Christian Schläger, Dept. of Neuroimmunology, Institute for MS Research, Göttingen, Germany

Thomas Skripuletz, Hannover Medical School, Germany

Volker Siffrin, Dept. of Neurology, University of Mainz, Germany

Friederike Vollmar, Dept. of Neurology, University of Würzburg, Germany

Claudia Wrzos, Dept. of Neurology, University of Göttingen, Germany

Hong Xu, Marburg, Dept. of Neurology, University of Marburg, Germany
